# Adherence to cardioprotective medications and mortality among patients with diabetes and ischemic heart disease

**DOI:** 10.1186/1471-2261-6-48

**Published:** 2006-12-15

**Authors:** P Michael Ho, David J Magid, Frederick A Masoudi, David L McClure, John S Rumsfeld

**Affiliations:** 1 Cardiology Section, Denver VA Medical Center, Denver, CO, USA; 2 Department of Medicine, University of Colorado Health Sciences Center, Denver, CO, USA; 3 Department of Biometrics and Preventive Medicine, University of Colorado Health Sciences Center, Denver, CO, USA; 4 Clinical Research Unit, Kaiser Permanente of Colorado, Denver, CO, USA; 5 Department of Medicine, Denver Health Medical Center, Denver, CO, USA

## Abstract

**Background:**

Patients with diabetes and ischemic heart disease (IHD) are at high risk for adverse cardiac outcomes. Clinical practice guidelines recommend multiple cardioprotective medications to reduce recurrent events. We evaluated the association between cardioprotective medication adherence and mortality among patients with diabetes and IHD.

**Methods:**

In a retrospective cohort study of 3,998 patients with diabetes and IHD, we evaluated use of ACE inhibitors or angiotensin receptor blockers, β-blockers, and statin medications. Receipt of cardioprotective medications was based on filled prescriptions. Medication adherence was calculated as the proportion of days covered (PDC) for filled prescriptions. The primary outcome of interest was all-cause mortality.

**Results:**

The majority of patients (92.8%) received at least 1 cardioprotective medication. Patients receiving any medications had lower unadjusted mortality rates compared to patients not receiving any medications (7.9% vs. 11.5%; p = 0.03). In multivariable analysis, receipt of any cardioprotective medication remained associated with lower all-cause mortality (OR 0.65; 95% CI 0.43–0.99). Among patients receiving cardioprotective medications, the majority (80.3%) were adherent (PDC ≥ 0.80). Adherent patients had lower unadjusted mortality rates (6.7% vs. 12.1%; p < 0.01). In multivariable analysis, medication adherence remained associated with lower all-cause mortality (OR 0.52; 95% CI 0.39–0.69) compared to non-adherence. In contrast, there was no mortality difference between patients receiving cardioprotective medications who were non-adherent compared to patients not receiving any medications (OR 1.01; 95% CI 0.64–1.61).

**Conclusion:**

In conclusion, medication adherence is associated with improved outcomes among patients with diabetes and IHD. Quality improvement interventions are needed to increase medication adherence in order for patients to maximize the benefit of cardioprotective medications.

## Background

Diabetes is prevalent and an important risk factor for the development of ischemic heart disease (IHD). Approximately 20% of patients with diabetes have IHD, and patients with both conditions are at particularly high risk of for adverse outcomes [1,2]. Practice guidelines recommend at least four pharmacologic agents to reduce the risk of adverse cardiac events in this population, including antiplatelet agents, β-blockers, HMG-CoA reductase inhibitors, and angiotensin-converting enzyme (ACE) inhibitors [3,4].

Prior studies suggest, however, that adherence to chronic cardioprotective medications is suboptimal, which may limit the potential benefits of these therapies [5]. In one study, only 21% of patients with IHD were consistently taking the combination of aspirin, β-blockers, and lipid-lowering therapy [6]. In addition, another study found that only 36% of IHD patients were still adherent with statin medications 2 years after the index prescription [7]. Prior studies, however, have not evaluated the association between cardioprotective medication adherence, and mortality among patients with diabetes and IHD.

Accordingly, the objective of this study was to evaluate the relationship between cardioprotective medication (i.e., angiotensin-converting enzyme inhibitors, β-blockers, and HMG-CoA reductase inhibitors) use and outcomes among patients with diabetes and IHD. Specifically, we assessed the association between receipt of 1 or more cardioprotective medications and mortality. Second, among patients receiving cardioprotective medications, we assessed the association between medication adherence and mortality. The findings of this study may have important implications for identifying gaps in the care of patients with diabetes and IHD, as well as the development of interventions to improve patient outcomes.

## Methods

### Study setting

Kaiser Permanente of Colorado (KPCO) is an integrated, nonprofit Managed Care Organization (MCO) that provides medical services to more than 400,000 members in the Denver, Colorado metropolitan area. A diabetes disease registry was established on September 17, 2002. Patients with diabetes who are 18 years of age or older are initially identified by an algorithm applied to KPCO automated databases consisting of pharmacy records (e.g., oral hypoglycemics, or insulin), laboratory data (e.g., hemoglobins A1C or glucose lab results), hospitalization records and outpatient diagnoses. Once a potential patient is identified, the diagnosis of diabetes is validated by chart review before inclusion in the registry.

### Patients

We conducted a retrospective cohort study of patients with ischemic heart disease enrolled in the KPCO diabetes registry. Patients who were in the registry as of September 17, 2002 and had continuous enrollment through December 31, 2003, were included. Receipt of cardioprotective medication(s) and medication adherence was assessed during calendar year 2003. The outcomes were ascertained from January 1, 2004 through April 30, 2005, which was the most recent date that follow-up data was available.

The diagnosis of ischemic heart disease was based on ICD-9 (International Classification of Diseases, 9^th ^Revision), CPT (Current Procedural Terminology) and/or DRG (Diagnosis-Related Group System) codes. We only included patients with a diagnosis of acute myocardial infarction based on ICD-9 or DRG codes, and/or underwent a coronary revascularization procedure (i.e., percutaneous coronary intervention or coronary artery bypass graft surgery). These criteria have been found to be highly specific for IHD [8]. Based on the above criteria, 3,998 patients were identified and comprise the cohort for the study.

Baseline patient demographics, co-morbidities, vital signs, and laboratory data were derived from the automated KPCO databases, including pharmacy records, laboratory data, hospitalization records and outpatient diagnoses. For laboratory data, the baseline LDL cholesterol and hemoglobin A1C levels were defined as the most current measurement during calendar year 2002. For blood pressure data, if 2 or more measurements were available in 2002, the two most recent measurements were averaged. Patients were followed until death or disenrollment from the health plan. As of April 30, 2005, 97.1% of the patients were still enrolled or had died.

### Medications

KPCO has an automated pharmacy that records all medications dispensed at each outpatient facility. Almost all patients, 98%, have prescription drug coverage for a nominal copayment. The nominal copayment and the convenience of pharmacies located at the same site as the healthcare provider's offices serve as incentives for patients to fill their prescriptions within the system.

### Cardioprotective medications

The receipt of 1 or more cardioprotective medication(s) was based on any filled prescriptions for angiotensin-converting enzyme (ACE) inhibitors, angiotensin receptor blockers (ARB), β-blockers, or HMG-CoA reductase inhibitors using the KPCO pharmacy records. Patients were categorized as having a cardioprotective medication if a prescription was filled for at least one of the indicated medications during calendar year 2003. In our primary analysis, we considered ARBs as an ACE-inhibitor equivalent medication. Therefore, patients were also categorized as having a cardioprotective medication if a prescription was filled for an ARB in calendar year 2003. Only 38 patients (<1%) in the entire cohort filled a prescription for an ARB and none of the other cardioprotective medications.

### Medication adherence

Medication adherence was calculated as the proportion of days covered (PDC), based on the total number of days supplied for filled prescriptions over the observation time interval [9]. We defined this time interval as a minimum of 90 days and maximum of 365 days to ensure stable estimates for refill adherence in our primary analysis. A PDC was derived for each of the classes of medications important to patients with diabetes and IHD, and recommended by national guidelines, including angiotensin-converting enzyme inhibitors or ARBs, β-blockers, and HMG-CoA reductase inhibitors (statins) [3,4]. For patients prescribed multiple classes of medications, a summary PDC was calculated based on the averaged PDC for all of the medications. In the primary analysis, patients were classified as 'adherent' based on a summary PDC ≥ 0.80, consistent with the dichotomization of medication adherence in the literature [10-16]. Applying this algorithm, a summary PDC was calculated for 3,696 patients with diabetes and IHD.

### Dependent or outcome variable

The primary outcome of interest was all-cause mortality during the follow-up period. Data on mortality was derived from the KPCO automated databases and validated by comparison with other internal KPCO data sources.

### Statistical analysis

Baseline demographic factors, co-morbidities, and the proportion of patients attaining A1C, BP and LDL guideline indicated levels were compared between patients receiving 1 or more versus none of the cardioprotective medications using the chi-square test for categorical variables and t-test for continuous variables. Next, the relationship between receipt of cardioprotective medications and all-cause mortality was evaluated using the chi-square test.

In multivariable analysis, logistic regression models were constructed to evaluate the independent association between receipt of 1 or more cardioprotective medication(s) and mortality. Models were built by applying backward regression (p < 0.10 to enter and p < 0.05 to remain in the model) to the variables listed in Table 1 and included patient demographic factors, co-morbidities, and baseline A1C, BP and LDL levels. Then, the receipt of any cardioprotective medication variable was entered into this baseline risk model to determine its association with mortality. Odds ratios and 95% confidence intervals were calculated for each independent variable in the multivariable models.

**Table 1:** Baseline characteristics of patients with diabetes and ischemic heart disease (n = 3,998).

**Variable**	**No cardioprotective medication **(n = 288)	**1 or more cardioprotective medication(s) **(n = 3710)	**p-value**
Male	50.7	61.6	<0.01
Age, mean (STD)	67.3(14.5)	69.3(9.8)	0.02
Hypertension	74.3	92.5	<0.01
Prior myocardial infarction	46.5	46.6	0.97
Peripheral vascular disease	8.0	10.2	0.24
Cerebrovascular disease	30.9	34.1	0.27
Chronic obstructive pulmonary disease	29.9	28.6	0.66
Heart failure	25.3	36.3	<0.01
Sleep apnea	20.1	23.4	0.21
Renal insufficiency	10.4	15.4	0.02
Retinopathy	6.9	6.6	0.82
Hypercholesterolemia	45.1	85.3	<0.01
Blood pressure <130/80 mm Hg at baseline	47.9	39.5	<0.01
LDL cholesterol <100 mg/dl at baseline	78.8	73.6	0.05
Hemoglobin A1C <7% at baseline	64.2	45.7	<0.01

Next, to evaluate the association between medication adherence and mortality, the cohort was restricted to patients receiving 1 or more cardioprotective medications (n = 3,696). Baseline characteristics were compared between adherent and non-adherent patients using the same statistical methods previously described. All-cause mortality was compared between the two patient adherence groups using the chi-square test. In multivariable analysis, logistic regression models were constructed in a similar fashion as the risk models for cardioprotective medications to evaluate the independent association between medication adherence and mortality.

In secondary analysis, we performed survival analyses and constructed multivariable Cox proportional hazards models to assess the relationship between receipt of 1 or more cardioprotective medication(s) and medication adherence with time to death due to any cause during the follow-up period. Hazards ratios and 95% confidence intervals were calculated for each independent variable in the multivariable models. Second, we evaluated the association between medication adherence and mortality for each individual class of medications. Third, we assessed whether there would be a gradient of benefit between medication adherence, medication non-adherence, and not receiving any cardioprotective medications by creating a three-level categorical variable and entered this variable into our multivariable models. The same multivariable modeling approach was used as the primary risk models.

To further assess the robustness of our findings, we performed several additional analyses. Since the ACC/AHA guidelines do not specifically mention angiotensin receptor blocker medications as an alternative to ACE-inhibitors, we excluded angiotensin receptor blocker medications from the analysis (3). Second, in separate regression models, we adjusted for the specific cardioprotective medication prescribed and also the total number of cardioprotective medications in the medication adherence risk models. Two-way interaction terms between the number of prescribed cardioprotective medications and medication adherence were also evaluated in the multivariable models. These interaction terms were not statistically significant and therefore are not presented further. Third, we re-defined the PDC by changing the minimal observation time interval window to 240 days. Fourth, we changed the dichotomization of medication adherence to a summary PDC ≥ 0.50, ≥ 0.60, ≥ 0.70, and ≥ 0.90. The analyses were repeated and multivariable regression models were constructed in a similar fashion as the primary risk models. The results of these additional analyses were consistent with our primary findings.

Finally, there were 14 patients who were prescribed at least 1 cardioprotective medication for less than 90 days during 2003. A 90-day minimum observation time interval window was chosen initially to ensure stable estimates for refill adherence. Therefore, in our primary analysis, these patients were categorized as receiving a cardioprotective medication, but they were excluded from the analysis on medication adherence. We also performed a series of sensitivity analyses to confirm our primary findings. For the cardioprotective medication analysis, we categorized these patients as not receiving a cardioprotective medication and also excluded these patients in separate analyses. For the medication adherence risk models, we categorized these patients as both adherent and non-adherent in separate analyses. The results of the sensitivity analysis were consistent with our primary findings.

The study was approved by the Kaiser Permanente Colorado Institutional Review Board. All analyses were performed using the SAS statistical package version 9.1 (SAS Institute, Cary, NC).

## Results

### Cardioprotective medications

Baseline characteristics of patients receiving any cardioprotective medication(s) versus none are listed in Table 1. The majority of patients (92.8%) received at least 1 or more cardioprotective medication(s). Among patients receiving any cardioprotective medications, 78.6% received 2 or more and 38.7% received all 3 guideline-indicated medications. Patients receiving cardioprotective medications were older and had more co-morbidities.

In unadjusted analysis, patients receiving any cardioprotective medication(s) had lower all-cause mortality (7.9% vs. 11.5%; p = 0.03) compared to patients receiving none of the medications. In multivariable logistic regression analysis, patients receiving 1 or more cardioprotective medication(s) had lower all-cause mortality (OR 0.65; 95% CI 0.43–0.99). Similarly, in Cox proportional hazards models, patients receiving any cardioprotective medication(s) remained at significantly lower risk for death during the follow-up period (HR 0.67; 95% CI 0.46–0.97).

### Medication adherence

Among patients receiving cardioprotective medications, baseline characteristics of adherent versus non-adherent patients are listed in Table 2. The majority of the patients (80.3%) were adherent, defined as a PDC ≥ 0.80. Adherent patients were older, but had fewer co-morbidities. They were more likely to be at LDL goal at baseline compared to non-adherent patients.

**Table 2:** Baseline characteristics of patients according to medication adherence (n = 3,696).

**Variable**	**Non-adherent **(n = 728)	**Adherent **(n = 2968)	**p-value**
Male	58.9	62.0	0.13
Age, mean (STD)	67.7(11.3)	69.6(9.5)	<0.01
Hypertension	91.8	92.3	0.59
Prior myocardial infarction	49.3	45.7	0.08
Peripheral vascular disease	11.8	9.5	0.07
Cerebrovascular disease	35.8	33.1	0.16
Chronic obstructive pulmonary disease	31.6	27.7	0.04
Heart failure	41.6	34.4	<0.01
Sleep apnea	25.0	22.8	0.21
Renal insufficiency	19.0	14.1	<0.01
Retinopathy	8.2	6.1	0.03
Hypercholesterolemia	81.5	85.5	<0.01
Statin prescription	66.7	75.1	<0.01
ACEI or ARB prescription	71.0	76.0	<0.01
β-blocker prescription	62.2	66.3	0.04
Blood pressure <130/80 mm Hg at baseline	39.3	39.5	0.91
LDL cholesterol <100 mg/dl at baseline	68.8	74.8	<0.01
Hemoglobin A1C <7% at baseline	46.1	45.9	0.88

ACEI: Angiotensin-converting enzyme inhibitor ARB: Angiotensin Receptor Blocker

During follow-up, adherent patients had lower unadjusted all-cause mortality rates (6.7% vs. 12.1%; p < 0.01). In multivariable logistic regression analysis, adherent patients remained at lower risk for all-cause mortality (OR 0.52; 95% CI 0.39–0.69) (Table 3). In Cox proportional hazards models, adherent patients also had a lower risk for death due to any cause during the follow-up period (HR 0.53; 95% CI 0.41–0.68).

**Table 3:** The association between medication adherence and mortality.

**Adherence measure**	**N**	**Percent adherent (PDC ≥ 0.80)**	**All-cause mortality**	
			
			**Unadjusted OR (95% CI)**	**Adjusted OR (95% CI)**
Summary measure	3696	80.3%	0.52 (0.40–0.68)	0.52 (0.39–0.69)
β-blocker medications	2440	76.6%	0.72 (0.52–1.00)	0.86 (0.61–1.22)
Statin medications	2833	81.9%	0.65 (0.45–0.94)	0.59 (0.41–0.87)
ACE-inhibitor medications	2379	80.0%	0.56 (0.40–0.80)	0.55 (0.38–0.78)

In secondary analysis of the individual classes of medications, adherence to statin (OR 0.59; 95% CI 0.42–0.87) and ACE-inhibitor (OR 0.55; 95% CI 0.38–0.78) medications were also associated with lower risk for all-cause mortality (Table 3). There was a trend for an association between adherence to β-blockers and lower mortality, however it was not statistically significant (OR 0.86; 95% CI 0.61–1.22). In addition, the association between medication adherence and mortality for the summary measure and the individual classes of medications was consistent using different PDC cut-off values to define adherence (Table 4).

**Table 4:** The association between medication adherence and mortality for different cut-offs of the PDC.

**PDC cut-off for adherence**	**Summary measure OR (95% CI)**	**B-blocker OR (95% CI)**	**Statin OR (95% CI)**	**ACE-inhibitor OR (95% CI)**
≥0.50	0.49 (0.30–0.80)	0.93 (0.55–1.56)	0.63 (0.34–1.15)	0.48 (0.28–0.81)
≥0.60	0.46 (0.31–0.67)	0.83 (0.55–1.26)	0.60 (0.37–0.98)	0.48 (0.31–0.73)
≥0.70	0.45 (0.33–0.61)	0.88 (0.61–1.28)	0.58 (0.38–0.88)	0.47 (0.32–0.69)
≥0.80	0.52 (0.39–0.69)	0.86 (0.61–1.22)	0.59 (0.41–0.87)	0.55 (0.38–0.78)
≥0.90	0.66 (0.51–0.86)	0.83 (0.60–1.14)	0.66 (0.47–0.93)	0.61 (0.43–0.85)

**Figure 1:**
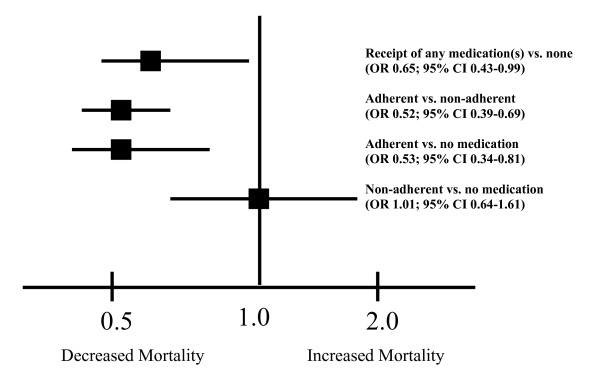
The association between receipt of cardioprotective medications, medication adherence and mortality.

Finally, compared to patients who did not receive any cardioprotective medications, patients who were adherent to medications had lower mortality risk (OR 0.53; 95% CI 0.34–0.81) (Figure 1). However, there was no difference in mortality between patients receiving cardioprotective medication who were non-adherent compared to patients who were not receiving any cardioprotective medications (OR 1.01; 95% CI 0.64–1.61).

## Discussion

The objective of this study was to evaluate the association between cardioprotective medication adherence and mortality among patients with diabetes and IHD. We found that patients receiving 1 or more cardioprotective medication(s) had lower all-cause mortality compared to patients receiving none of the medications. In addition, among those receiving cardioprotective medication(s), adherent patients were at lower risk for all-cause mortality compared to non-adherent patients. In contrast, patients receiving cardioprotective medications who were non-adherent had similar mortality risk to patients receiving none of the cardioprotective medications. These findings suggest that current quality improvement efforts should be expanded to include the assessment of medication adherence as a key component of secondary prevention care in addition to the prescription of indicated medications.

Clinical trials have demonstrated the efficacy of medications such as β-blockers, ACE inhibitors, and statin medications [3,17,18]. However, the effectiveness of combinations of these medications remains less well defined among patients with chronic IHD. Prior studies have demonstrated a mortality benefit among high-risk IHD patients prescribed combinations of cardioprotective medications after acute coronary syndrome presentation [19,20]. However, among patients with IHD and diabetes, only 11% of patients were prescribed the combination of antiplatelet agent, ACE-inhibitor, and statin medications [21]. In our study, 78.6% of patients were receiving 2 or more medications and 38.7% were receiving all 3 cardioprotective medications. In addition, patients receiving any cardioprotective medication had lower mortality compared to those receiving none of the medications. Our findings demonstrate the effectiveness for combinations of cardioprotective medications and are consistent with national guideline recommendations that patients with diabetes and IHD should receive all of these therapies unless contraindications exist.

Studies evaluating medication adherence using pharmacy refill records among IHD patients suggest that non-adherence is a common phenomenon, but have typically focused on a single medication, and/or have not assessed the impact of non-adherence on outcomes. Wei, et al. previously demonstrated that patients adherent to β-blockers or statins had better outcomes compared to patients who were not prescribed either of these medications [14,15]. Blackburn, et al. found that patients adherent to statin medications were also more likely to adhere to β-blockers and ACE inhibitors. However, at 2-years from the index statin prescription, only 36.1% of patients with chronic IHD were still adherent with statins [22]. In our study, the proportion of adherent patients (80.3%) was higher than in other populations; and adherent patients had lower all-cause mortality compared to either non-adherent patients or patients not receiving any cardioprotective medications. These findings suggest that medication adherence should be routinely assessed and once identified, clinicians should approach medication non-adherence like an elevated systolic blood pressure reading or high LDL cholesterol, a risk marker for adverse outcomes that requires treatment and follow-up.

The association between medication adherence and mortality is likely mediated through direct and indirect mechanisms. In clinical trials, both statin and ACE-inhibitor medications have been shown to benefit patients with IHD, diabetes or both of these conditions [3]). The magnitude of mortality benefit seen in our study, however, was greater than the benefit demonstrated in clinical trials of these medications suggesting that medication adherence may also be correlated with self-care behaviors that are directly or indirectly related to outcomes [23-26]. For example, medication adherence has been associated with better outcomes regardless of treatment assignment in randomized controlled clinical trials [16,26-28]. Adherent patients may be more likely to follow lifestyle recommendations and other healthy behaviors leading to improved outcomes. Regardless of the mechanism, interventions are needed to improve adherence to medications as well as healthy lifestyle behaviors among patients with diabetes and IHD.

There was a trend for benefit of β-blockers adherence and improved outcomes among patients with diabetes and IHD, however it was not statistically significant. β-blockers have been shown to improve survival among patients presenting with an acute myocardial infarction and after recent ST elevation myocardial infarction (STEMI) [29]. In contrast, the benefit of β-blockers in patients with stable angina without prior MI or among those with a remote MI is less well defined. In a meta-analysis of randomized trials of patients with stable angina, there was no benefit of β-blockers compared to calcium channel blockers in reducing the composite endpoint of death or MI (OR 1.06; 95% CI 0.73–1.54) [3]. In our study, less than 50% of patients with IHD had a prior MI. In addition, because of our administrative data source, we cannot be certain about the timing of the prior MI event. These factors may have biased our results toward the null and the lack of a statistically significant association between β-blockers adherence and mortality. Nevertheless, β-blockers are effective antianginal medications and recommended by national guidelines for patients with chronic stable angina [3].

Several potential limitations of this study should be addressed. First, this was a single MCO study using administrative data and we did not have data on cause of death. However, it was a population-based study of a large integrated healthcare delivery organization. Second, our inclusion criteria may not have identified all patients with IHD in the diabetes registry, however, the criteria used are highly specific and have a high positive predictive value for identifying patients with IHD [8]. Third, adherence was based on pharmacy refill records. However, 98% of the patients had prescription drug coverage and the small proportion of missing data would, if anything, tend to bias the results toward the null. Pharmacy refill records have been found to be highly correlated with electronic adherence monitoring and the act of refilling a medication is the first step towards taking a medication, reflecting the patients' active decision to continue with therapy [30]). Fourth, the categorization of adherent patients based on a PDC ≥ 0.80 versus <0.80 was somewhat arbitrary. This dichotomization of adherence, however, was consistent with the literature [10-16]. Furthermore, we examined the effect of medication adherence on outcomes using multiple different cut-points for dichotomizing adherence (i.e., PDC ≥ 0.50, ≥ 0.60, ≥ 0.70, or ≥ 0.90). Regardless of the cut-point, the association between adherence and improved outcomes was consistent. Fifth, we did not assess for specific contraindications to medications. However, our definition for receipt of any cardioprotective medication(s) was broadly defined and patients only had to receive any one of four indicated medications for patients with diabetes and IHD. Future studies should assess the reasons why patients did not receive the indicated cardioprotective medication(s) and also determine if overall pill burden impacts medication adherence. Finally, we did not assess adherence to aspirin because it is often obtained over the counter or adherence to thienopyridines because it is a class IIa recommendation for secondary prevention among patients with chronic CAD. Our main focus was on class I indicated medications, including ACE-inhibitors, β-blockers, and statin, for patients with CAD and diabetes.

## Conclusion

We found that among those receiving cardioprotective medication(s), adherent patients were at lower risk for all-cause mortality compared to non-adherent patients. In contrast, patients receiving cardioprotective medications who were non-adherent had similar mortality risk to patients receiving none of the cardioprotective medications. Our findings suggest that medication non-adherence should be routinely assessed and once identified, clinicians should engage patients in a discussion of the reasons for non-adherence and promote ways of improving adherence to prescribed medications. Finally, quality improvement interventions are needed to increase medication adherence among patients with diabetes and IHD to maximize the benefit of cardioprotective medications.

## Competing interests

The author(s) declare that they have no competing interests.

## Authors' contributions

All authors made substantial contributions to conception and design, or acquisition of data, or analysis and interpretation of data; PMH, DJM, FAM, JSR have been involved in drafting of manuscript or revising it critically for important intellectual content; and all authors have given final approval of the version to be published.

## Pre-publication history

The pre-publication history for this paper can be accessed here:


